# Telemetric monitoring of 24 h intraocular pressure in conscious and freely moving C57BL/6J and CBA/CaJ mice

**Published:** 2008-04-18

**Authors:** Ruixia Li, John H.K. Liu

**Affiliations:** Hamilton Glaucoma Center and Department of Ophthalmology, University of California, San Diego, La Jolla, CA

## Abstract

**Purpose:**

To study change patterns of 24 h intraocular pressure (IOP) in conscious and freely moving mice using telemetry.

**Methods:**

Adult C57BL/6J and CBA/CaJ mice were entrained to a standard 12 h light and 12 h dark cycle. A telemetric pressure transmitter was implanted subcutaneously on the upper back of each light-dark entrained mouse, and the pressure catheter tip was inserted into the vitreous chamber. Broadcasted IOP data were received at 120 Hz. Means of 2 min IOP were recorded every 5 min for 4–13 days to generate the 24 h IOP pattern in each mouse strain. The pattern of IOP in the C57BL/6J strain was also determined under an acute constant dark condition for 24 h.

**Results:**

There were distinct patterns of 24 h IOP in the C57BL/6J and CBA/CaJ mouse strains. Under the standard light-dark condition, IOP was higher during the dark period than the light period in both strains. Elevation in IOP from the light period to the dark period was significantly smaller in the CBA/CaJ strain (1.6±1.7 mmHg, mean± standard deviation (SD), n=21) than in the C57BL/6J strain (3.4±2.5 mmHg, n=20). The 24 h IOP pattern in the C57BL/6J strain persisted under an acute constant dark condition (n=8).

**Conclusions:**

Distinct change patterns of 24 h IOP appeared in these two mouse strains. Although mean IOP during the dark period was significantly higher than the light period in both strains, the magnitudes of light-dark IOP elevation differed. The 24 h IOP change pattern can be driven endogenously in the absence of light.

## Introduction

Daily variation of intraocular pressure (IOP) is important information for the diagnosis and treatment of glaucoma [1,2]. To determine IOP variation, continuous IOP monitoring under unrestrained conditions offers significant advantages over periodic IOP measurements under restrained conditions. At the present time, no clinical technique is available to monitor human IOP continuously. However, continuous IOP monitoring has been successfully demonstrated in conscious and freely moving laboratory rabbits using telemetry [3-8]. The telemetric pattern of 24 h IOP in rabbits agrees with the 24 h IOP pattern observed in conscious, restrained rabbits using a pneumatonometer [9,10].

Increasing applications of genetic technology for studying IOP [11-13] have raised interest in the daily IOP variation in mice [14-17]. Until recently, change patterns of 24 h IOP in this nocturnally active rodent have been obtained using periodic IOP measurements involving microneedle cannulations of the anterior chamber under general anesthesia [18]. The 24 h IOP change pattern in conscious and freely moving mice is not known. In the present study, telemetry with a miniaturized pressure transmitter was used to study the 24 h IOP patterns in two inbred mouse strains. One was the widely used C57BL/6J strain. The other was the CBA/CaJ strain that may not have had significant IOP variation between the light and dark periods compared to many other mouse strains including the C57BL/6J strain [14].

## Methods

Adult C57BL/6J and CBA/CaJ mice (20–25 g) were obtained from the Jackson Laboratory (Bar Harbor, ME) and entrained to a daily 12 h light (6 AM to 6 PM) and 12 h dark cycle for at least two weeks before the experiments. Food and water were freely available, and the housing temperature was constant at 22 °C. Experiments complied with the guidelines of the Institute for Laboratory Animal Research, and the procedures were approved by the Institutional Animal Care and Use Committee.

A battery-powered, telemetric pressure transmitter (Model PA-C20, weight 3.4 g; Data Sciences International, St. Paul, MN) was implanted subcutaneously on the upper back under aseptic conditions. The mouse was anesthetized with intramuscular ketamine (100 mg/kg) and xylazine (10 mg/kg). A midline incision was made to the dorsal neck. Subcutaneous tissues toward the upper back were gently separated to form a packet for placing the pressure transmitter that was immobilized with sutures to the skin. A subcutaneous fistula was created from the dorsal neck to the temporal eyelid for routing the pressure catheter. Guided by a 20 gauge needle bevel, the catheter tip (0.3–0.4 mm external diameter) was inserted halfway between the limbus and the optic nerve bundle into the vitreous chamber. The catheter tip was advanced 2 mm into the vitreous chamber, passing the pupillary midpoint. The eyeball diameter was 3.5–4 mm. Anchoring the distal pressure catheter on the thin sclera was impractical. The distal pressure catheter was placed inside a 5 mm polyethylene tubing (0.4 mm internal diameter; Technicon, Tarytown, NY), and the tubing was glued to the parietal bone and zygomatic arch using tissue adhesive (Loctite 454 gel; ALZET, Cupertino, CA). The proximal pressure catheter was looped and internalized at the dorsal neck, and the incision was closed with sutures. Neosporin ointment was applied over the surgical areas. For each mouse, the procedure was only performed on one eye. The postoperative mouse was allowed to recover in an individual cage under the standard 12 h light and 12-h dark cycle. Tetracycline was added to the drinking water (3 mg/ml) for 24 h.

The transmitter broadcasted radio frequency pressure information. Since the pressure catheter tip was in the vitreous, the vertical distance between the vitreous and the transmitter placed in the upper back created a hydrostatic pressure in the recording system. A pressure artifact related to this hydrostatic pressure could have been significant when the posture changed [19] such as during feeding or drinking from resources placed on the overhead steel grid or when climbing upside down on the grid. To minimize the pressure artifact, the overhead grid was removed and the food and water resources were placed on the cage floor during data collection.

**Figure 1:**
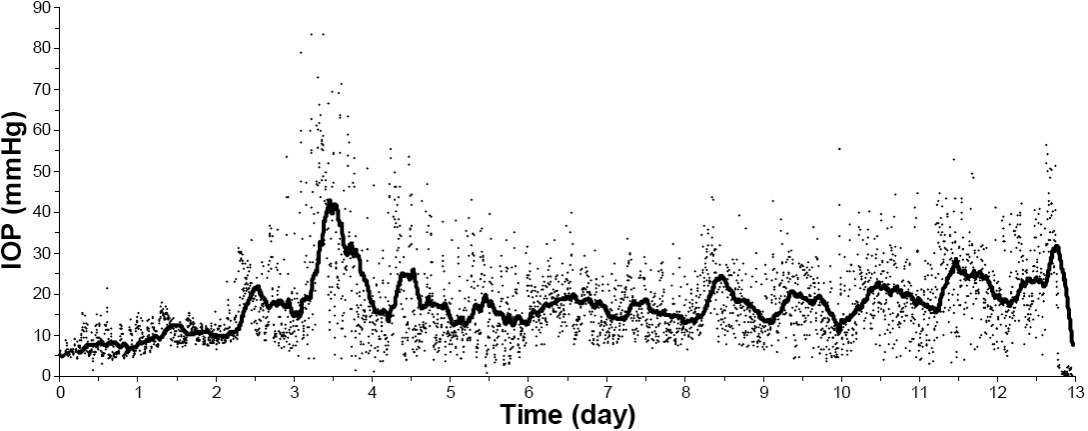
Continuous 13 day record of intraocular pressure (IOP) in one conscious and freely moving C57BL/6J mouse. Recording started at noon under the 12 h light (6 AM to 6 PM) and 12 h dark cycle. Each dot represents a 2 min IOP mean for a 5 min interval. The trend-line was determined using the moving average of 72 data points. The recording system failed near the end of 13 days. A spontaneous IOP fluctuation seen on day 4 in this mouse could appear anytime during the data collection periods in other mice.

The receiver for the telemetric pressure information was placed under the cage. The software was programmed to receive pressure information at 120 Hz. Continuous IOP data received could be viewed online, and pulsations of IOP due to the heart beats were used to verify overall success in the surgical procedure and telemetric recording. The software was also programmed to average pressure readings for 2 min (14,400 readings) every 5 min and to continuously archive the pressure average. Cage changes and incidental tasks were performed during the 3 min time break. Data from two mice in separate cages could be processed in sequence every 5 min. Immediately after the surgery, IOP might be abnormally high or low due to the insertion of the pressure catheter tip or the leakage of vitreous humor. It usually took 24–48 h to start showing a 24 h IOP pattern. Data from this initial adjustment period were not used for analyses. After the initial adjustment period, manual handling of the postoperative mouse was avoided whenever possible.

Data were collected from 20 C57BL/6J mice for 5–13 days under the standard 12 h light and 12 h dark cycle until the pressure recording system failed. Long-term data collections beyond two weeks were not successful because of a breakup of the subcutaneous packet housing the pressure transmitter. For a successful recording of one light-dark cycle, 144 means of 2 min IOP during the light period and 144 means during the dark period were recorded. For 8 of these 20 postoperative mice, additional data were collected for 24 h under an acute constant dark condition after a consistent 24 h IOP pattern had been established. The purpose was to evaluate whether or not a 24 h IOP change pattern was driven by light exposure. To provide an acute constant dark condition, the lights-on switch at 6 AM was disabled. The telemetric data were continuously collected in the 12 h subjective light period and the 12 h dark period. Data were collected from 21 CBA/CaJ mice for 5–13 days under the standard light-dark condition following the same experimental protocol.

Five C57BL/6J mice underwent the surgical procedure for implanting the pressure transmitter and routing the pressure catheter to the temporal eyelid, but the catheter tip was not inserted into the vitreous chamber. Instead, the catheter tip was placed outside the eyeball unsealed to monitor the basal pressure pattern in the recording system. Telemetric data were collected in these control experiments for 4–10 days as described previously.

For quantitative data analyses, the hourly pressure averages around the clock were calculated first for each mouse using the complete data collected under the same experimental condition. Then, the 24 h change pattern of pressure under each experimental condition was determined by summarizing the hourly pressure averages from all the mice in the group (n=5–21). Repeated measures ANOVA was used to determine the presence of a difference in these hourly pressure averages for the experimental group. Statistical comparisons of the average pressures between the light and dark periods were performed using the paired *t*-test. Inter-strain comparisons were performed using the Student’s *t*-test. A p<0.05 was regarded as statistically significant.

**Figure 2:**
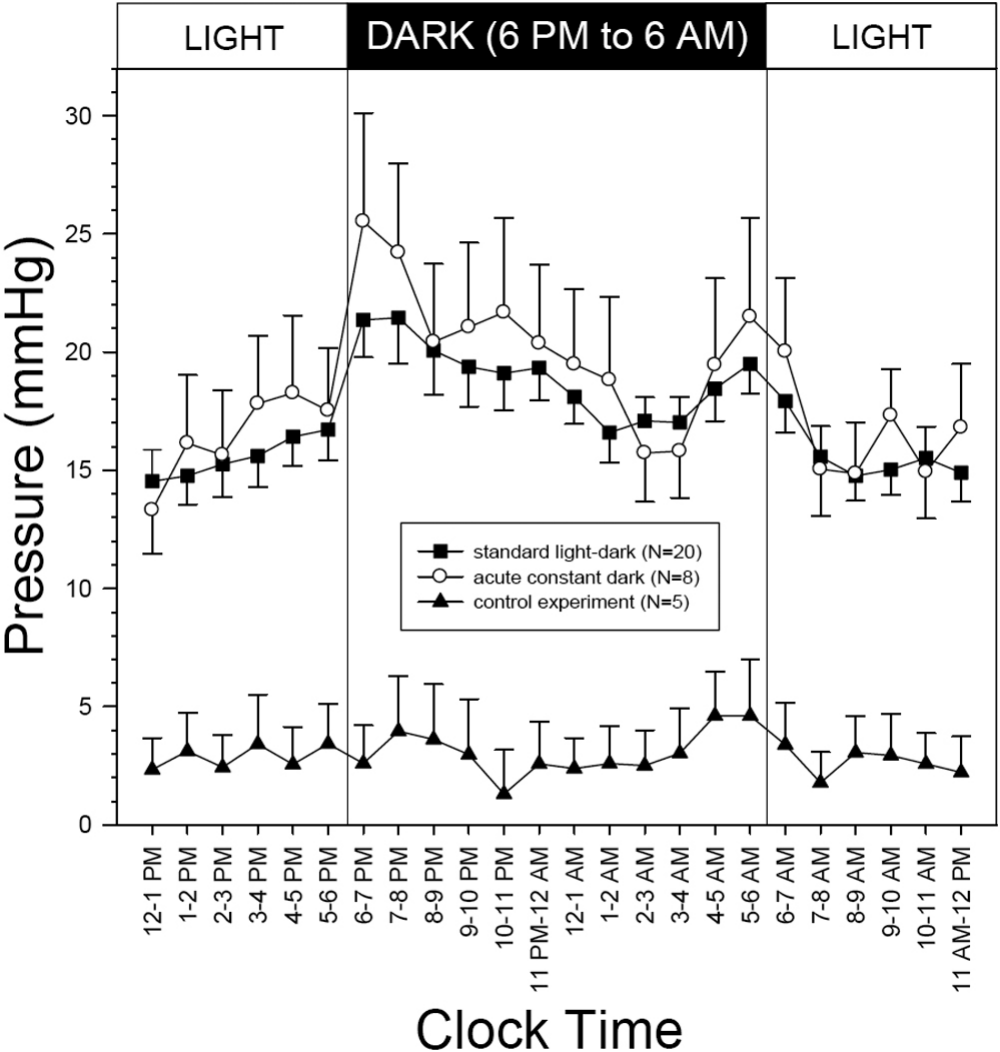
Change pattern of 24 h intraocular pressure (IOP) in C57BL/6J mice. Error bars represent SEM. Repeated measures ANOVA indicated a statistical difference in the hourly IOP averages under the standard light-dark and the acute constant dark conditions but not in the control experiment.

## Results

Telemetric IOP monitoring in mice was challenging due to the small size of the eyeball. Complications were encountered most frequently on the insertion of the pressure catheter tip into the vitreous chamber and during the first two postoperative days. Significant hemorrhaging during surgery usually caused a blockage of the pressure catheter. A persistent ocular inflammation/irritation could make the implanted pressure catheter tip uncomfortable to the host, and frequent eyeball scratching easily broke the setup. These complications led to no verifiable IOP pulsations online that corresponded to the heart beats. The overall success rate for continuous IOP recordings was about 40% (41/98) in the present study. In the successful recordings, there were minor adverse effects of the implantation to the locomotive activities because of an extra load of the pressure transmitter to the bodyweight. However, there was no alteration in the light/sleep and dark/active behavior patterns.

A complete pressure recording in one C57BL/6J mouse is presented in Figure 1. For this mouse, IOP was relatively low and flat shortly after the surgery and there was no identifiable 24 h pattern for approximately 48 h. A consistent 24 h IOP rhythm appeared after three days and up to 13 days, and significant IOP fluctuations occurred among the 2 min IOP means. In most postoperative eyes, the 2 min IOP fluctuations occurred while normal blinking and eye movement were lessened by the implanted pressure catheter. The spontaneous day-to-day fluctuation in IOP such as that on day 4 in Figure 1 could appear anytime during the data collection period.

Figure 2 summarizes the 24 h patterns of hourly pressure averages in the C57BL/6J mouse strain under three experimental conditions. Repeated measures ANOVA indicated a statistically significant variation in the hourly IOP averages under the standard light-dark and the acute constant dark conditions (p<0.001) but not in the control experiments (p=0.108). Hourly IOP was in general higher during the dark period than during the light period under the standard light-dark condition. There were two time-dependent IOP rises, one at the beginning of the dark period and the other at the transition from the dark period to the light period. The average IOP during the light period was 15.6±5.2 mmHg (mean±SD, n=20), and the average IOP during the dark period was 19.0±5.6 mmHg. The light-dark IOP difference of 3.4±2.5 mmHg was statistically significant (p<0.001). Figure 2 also shows a similar 24 h IOP change pattern under an acute constant dark condition (n=8). In the five control experiments with the pressure catheter tip placed outside the eyeball, the pressure registered at the transmitter was relatively flat throughout the 24-h cycle. The pressure difference of 0.3±1.5 mmHg between the light period (2.8±3.4 mmHg) and the dark period (3.1±4.1 mmHg) was not statistically significant.

**Figure 3:**
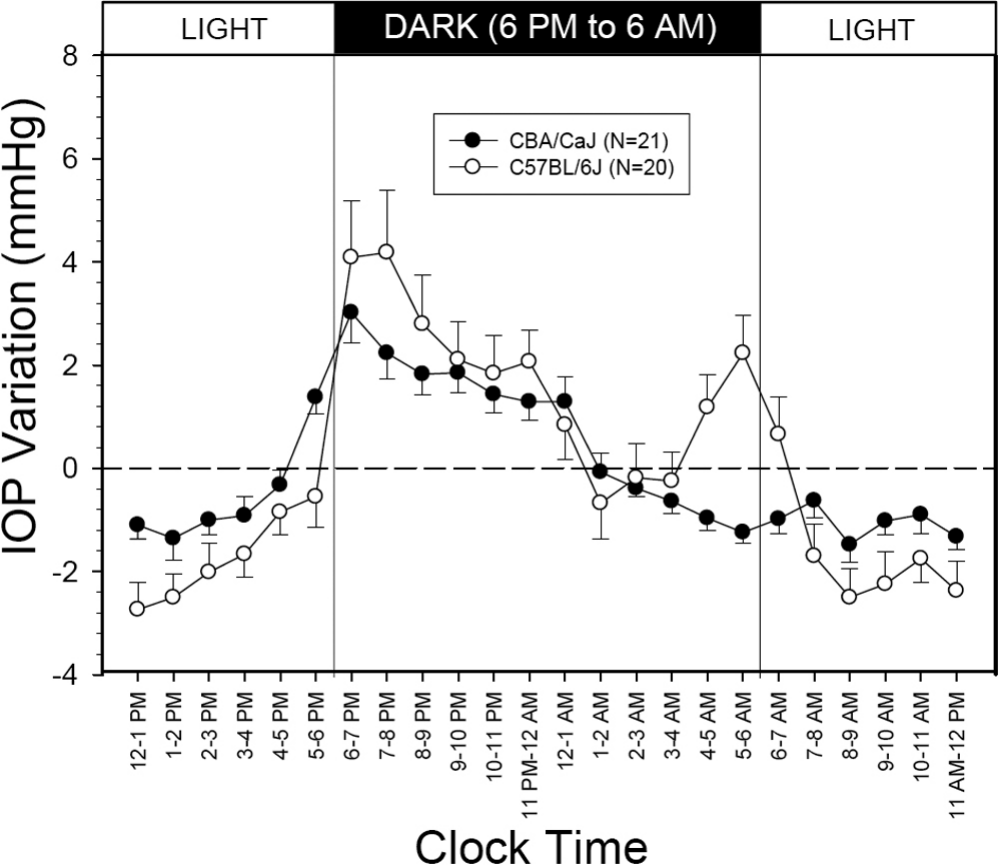
Variations of intraocular pressure (IOP) from the 24 h means in CBA/CaJ and C57BL/6J mice. Error bars represent SEM. Dashed line represents the zero variation from the 24 h IOP mean. A time-dependent IOP rise during the period of 4–7 PM only appeared in the C57BL/6J strain.

Twenty-one postoperative CBA/CaJ mice were housed under the standard 12 h light and 12 h dark cycle, and the change pattern of 24 h IOP was determined. A statistically significant variation in the hourly IOP averages occurred (p<0.001; repeated measures ANOVA). The average IOP was 12.4±2.0 mmHg during the light period and 14.0±2.5 mmHg during the dark period. The IOP difference between the light and dark periods (1.6±1.7 mmHg) was statistically significant (p=0.024). This intra-strain IOP difference between the light and dark periods in the CBA/CaJ mouse was significantly less than the intra-strain IOP difference in the C57BL/6J mouse (p=0.012). Variations in the hourly IOP from the 24 h means in the CBA/CaJ and C57BL/6J mouse strains are presented in Figure 3. In the CBA/CaJ mouse strain, there was only one time-dependent IOP rise at the beginning of the dark period whereas in the C57BL/6J mouse strain, there were two IOP rises during the 24 h period.

## Discussion

Telemetric IOP monitoring in conscious and freely moving mice offers an alternative to the currently available tonometric methods in mice. Telemetric IOP data can be collected continuously from mice in the absence of general anesthesia [18,20] or any other restrained condition [21-25]. The control experiments on C57BL/6J mice showed relatively stable hourly pressure averages in the recording system. The average pressure difference between the light and dark periods was about 0.3 mmHg in these experiments. When the pressure catheter tip was placed in the vitreous chamber, the 24 h IOP pattern was quite different from the control experiment and the light-dark pressure difference was much larger (3.4 mmHg). This rise and fall pattern of 24 h IOP in the C57BL/6J mouse strain persisted for 24 h under an acute constant dark condition, confirming that the 24 h IOP change pattern was not driven by light perception and was probably driven endogenously by clock genes in the suprachiasmatic nucleus [16]. The 24 h IOP change pattern might subside under a long-term constant dark condition [17], which was not evaluated in the present study due to the two-week limit on the recording time.

The CBA/CaJ mouse strain surprisingly showed a light-dark IOP difference [14]. However, the magnitude of light-dark IOP difference was statistically smaller in the CBA/CaJ strain than in the C57BL/6J strain, which was partially due to the absence of an IOP rise at the end of the dark period. In a previous study on 24 h pattern of total aqueous humor protein concentration, no difference was found between the C57BL/6J and CBA/CaJ mouse strains [26]. These results suggest that the 24 h rhythms of IOP and total protein concentration in the aqueous humor are not closely related parameters in mice.

We found that the light-dark IOP difference was 3.4 mmHg in the C57BL/6J mouse strain, which was close to the observation using the microneedle method under general anesthesia [16]. Thus, the gross light-dark IOP change pattern in conscious and freely moving mice can be approximated using an invasive tonometry under general anesthesia [15-17]. However, IOP telemetry enabled the pressure data to be sampled at a much higher rate than any other tonometric method. Continuous IOP monitoring can readily provide hour-by-hour IOP information as shown in the present study. Consequently, one is able to detect the small difference in the 24 h IOP change patterns between the C57BL/6J and CBA/CaJ mouse strains.

The minute-to-minute IOP fluctuation and the day-to-day spontaneous IOP fluctuation in conscious and freely moving mice (Figure 1) were much larger than the IOP fluctuations previously observed by periodic IOP measurements in anesthetized mice [15-17]. As demonstrated in the telemetric IOP monitoring in rabbits [4], restriction of physical activities during a regular tonometry could dampen the real IOP fluctuation. Fluctuation of IOP in a real life situation is probably more than what can be estimated using a non-continuous tonometry in any species. However, whether or not the large IOP fluctuation observed using telemetry in conscious and freely moving mice is free of artifacts cannot be thoroughly evaluated since there are no other publications in the literature for a comparison.

There are limitations of using telemetry to study IOP in mice. Accuracy of each pressure transmitter to the atmosphere pressure was verified as within ±3 mmHg of the manufacturer’s calibration before and after each experiment. Since the pressure transmitter was designed to monitor a wide range of blood pressure (−20–300 mmHg), a manufacturer’s accuracy limit of ±3 mmHg was not an issue for monitoring blood pressure. For IOP monitoring, this accuracy in pressure calibration had its consequences despite the reliability of the pressure recording. A distinct change pattern of 24 h IOP for a specific mouse strain can be considered valid, but the absolute IOP values may not be as accurate. This limitation on accuracy affected the IOP level for each mouse studied and the group means of the C57BL/6J and CBA/CaJ strains. The mean pressure in the control experiments was likely affected as well. Therefore, an accurate IOP difference between the CBA/CaJ strain and the C57BL/6J strain may need to be determined using the microneedle method [14]. On the other hand, the rise and fall of 24 h IOP from the 24-h mean in Figure 3 should not be affected significantly by the inaccuracy in the absolute IOP value.

Using the telemetric IOP model for pharmacological experiments in mice has additional limitations. After inserting the pressure catheter tip into the vitreous chamber, the barrier to ocular delivery of a test agent is no longer intact [27]. Bioavailability of a test agent may also be significantly affected by local tissue reactions to the implantation in the mouse eye. Considering these limitations, we conclude that the telemetric IOP monitoring in conscious and freely moving mice is a useful technique with restrictions. The technique is valid if the goal is to study time-dependent IOP change patterns such as the rise and fall during a circadian cycle. The validity is uncertain if the experimental result depends on absolute IOP values or ocular delivery to an intact eyeball.
